# The Maastricht Acute Stress Test (MAST): Physiological and Subjective Responses in Anticipation, and Post-stress

**DOI:** 10.3389/fpsyg.2017.00567

**Published:** 2017-04-19

**Authors:** Alexandra L. Shilton, Robin Laycock, Sheila G. Crewther

**Affiliations:** ^1^Department of Psychology and Counselling, School of Psychology and Public Health, La Trobe University, MelbourneVIC, Australia; ^2^School of Health and Biomedical Sciences, RMIT University, MelbourneVIC, Australia

**Keywords:** acute stress, MAST, sympatho-adrenal-medullary (SAM) axis, blood pressure, anxiety

## Abstract

The Maastricht Acute Stress Test (MAST) is designed to be a simple, quick, and non-invasive procedure aimed at activating the human stress system. The MAST has been developed by combining elements from two of the most common experimental paradigms measuring stress, the Trier Social Stress Test and the Cold Pressor Test. The aim of this study was to use the MAST procedure to elicit strong autonomic and subjective stress responses that can be quantified in terms of (systolic and diastolic) blood pressure, pulse rate (PR), and state anxiety ratings. In healthy individuals, the MAST induced a significant elevation of systolic blood pressure (SBP) from baseline for up to 30 min post-MAST, while diastolic blood pressure (DBP) dropped to baseline within 10 min post-MAST. Interestingly, the presentation of instructions alerting participants to the procedure resulted in significant elevation of both SBP and DBP above baseline. However, BP measurements prior to test initiation were not as high as those measured immediately after the MAST procedure. PR data showed limited variability across time points. Self-reported state anxiety increased dramatically from baseline to immediately following the MAST procedure. Further, individuals who reported higher levels of depression and stress were more likely to demonstrate larger increases in SBP in response to the MAST. Together, these results support the use of the MAST as a useful tool to activate both acute physiological and subjective measures of the stress response in healthy adults lasting up to 30 min.

## Introduction

The human stress response has an important adaptive role in everyday life, sometimes functioning to benefit the individual and sometimes producing maladaptive responses. Stress responses involve physiology, perception, emotion, and behavior (McEwen, 2008; Feder et al., 2009; Lupien et al., 2009). Physiologically, the maintenance and restoration of homeostasis during stressful events involves the activation and control of the neuroendocrine and autonomic stress systems (Ulrich-Lai and Herman, 2009). Stress activates the sympathetic branch [i.e., sympatho-adrenal-medullary (SAM) axis] of the autonomic nervous system (ANS) and gives rise to the well-known fight-or-flight response, which produces an increase in levels of adrenalin and noradrenalin, that affects blood pressure (BP), heart rate and respiration rate, while the opposite action of the parasympathetic branch counteracts such a response (Ulrich-Lai and Herman, 2009). The hypothalamic-pituitary-adrenal (HPA) axis also plays an important role in the peripheral physiological stress response through the release of glucocorticoids (i.e., cortisol in humans) into the bloodstream.

Several laboratory stress protocols have been developed to activate the human stress system, including the Cold Pressor Test (CPT) and the Trier Social Stress Test (TSST), and are designed to emulate acute, one-off stressors that occur in daily life (e.g., public speaking). However, there is great variability in the degree to which these experimental stressors, are capable of activating the ANS (in particular the SAM axis that is often monitored by BP) (see Smeets et al., 2012) in a lab situation and hence out of a real-life context. One hypothesis given for such variation is that the type of stressor to which a person is exposed, whether physical (e.g., pain, heat/cold) or psychological (e.g., public speaking, arithmetic task) in nature, has significantly different impacts on the physiological stress response of different individuals (Smeets et al., 2012). Early research suggested that physical stressors explicitly activate the sympathetic-adrenal system (Lundberg and Frankenhaeuser, 1980). However, later researchers argue that psychosocial stressors can equally elicit a response of the SAM axis (Skoluda et al., 2015). Various stress tests may also differ in their autonomic responses due to the effects of anticipatory fear. While anticipation of a psychosocial stressor (evaluated speech) has been reported to increase heart rate in women with higher levels of trait anxiety (Gonzalez-Bono et al., 2002), anticipation of physical stressors involving pain has been observed to lead to a reduction in heart rate in both human and animal samples (see Alm, 2004 for review). Furthermore, although there are limited studies on the anticipatory effects on BP, Marshall et al. (2002) demonstrated that systolic blood pressure (SBP), but not diastolic blood pressure (DBP), significantly increased from baseline after being told a blood test was imminent, suggesting that activation of the SAM axis may differ depending on the type of stressor, pre-existing levels of anxiety (and depression), and the timing of the measure relative to the stressor.

In seeking ecological validity many laboratory stress tests appear to better model moderate acute stress in daily life (e.g., public speaking; stressful work deadline), rather than overwhelming acute trauma (e.g., sexual assault) and longer-term chronic stress (e.g., childhood maltreatment and neglect, or low socioeconomic status). Nevertheless, certain stress tests appear to be more closely related to real-life stress than others. For example, one study showed heart rate reactivity to the laboratory CPT were related to heart rate reactivity in a real-life situation (giving a class presentation at university), while all other laboratory stress tests (i.e., cognitive tasks, social problem solving task) were not shown to relate to real-life hear rate reactivity (Johnston et al., 2008). Finally, there is also evidence to suggest that real or perceived lack of control during acute stress is more likely to negatively impact on behavior and performance (Glass et al., 1971), and is likely a key factor in real-life stress.

A more recent laboratory test that attempts to combine physical and psychological stress components (Smeets et al., 2012) has recently been developed to facilitate quantification of the human stress system responses including the SAM axis. The Maastricht Acute Stress Test (MAST) has been shown to elicit robust autonomic, glucocorticoid and subjectively reported psychological stress responses (Smeets et al., 2012), however, to date anticipatory stress responses have not been measured. The MAST procedure combines the most stressful features from two of the most common experimental paradigms, the TSST (involving novelty, unpredictability, ego involvement) and the CPT (involving physical pain). In direct comparison to a range of other validated stress protocols, including the TSST, CPT, Socially Evaluated Cold Pressor Test (SECPT), as well as a prolonged version of the SECPT, the MAST induced similar if not greater changes in BP immediately and 5 min following the conclusion of the stress test, and significant increases following the procedure in subjective experiences of stress, pain, and unpleasantness as measured on Visual Analog Scales (VASs) (Smeets et al., 2012). In addition, the procedure has incorporated lack of control by not allowing participants to know how long their hand will be submerged in water in each trial (see Materials and Methods), and is one key advantage of this procedure over the CPT, and thus the MAST, if post- stress responses are shown to last a sufficient duration, may provide a useful lab technique to assess cognition or attention under stress.

Cardiovascular reactivity to acute stress tests has been associated with pre-existing (clinical but also sub-clinical) symptoms of anxiety and depression, however, there is a great deal of variability across these studies. For example, individuals with high depressive (but non-clinical) symptoms demonstrate exaggerated BP and heart rate responses following a range of acute stress tests (e.g., Stroop test, speech, anger recall) (Light et al., 1998; Kibler and Ma, 2004), though the nature of the task may determine whether this relationship is shown (Yuenyongchaiwat et al., 2016). State anxiety has also been found to be positively associated with BP responses to the cold pressor and anger recall tests (Pointer et al., 2011), whilst subjectively perceived stress of a psychological stressor is also greater in those with higher anxiety and depressive symptoms (de Rooij et al., 2010). Conversely, other studies have shown a blunted cardiovascular response to acute stress tests [e.g., Stroop, mental arithmetic, paced auditory serial addition test (PASAT)] in individuals with high depressive symptoms (Chida and Hamer, 2008; Phillips et al., 2011).

Although the MAST has been utilized to assess the effects on cortisol and subjective levels of stress, affect, and anxiety (Smeets et al., 2012; Meyer et al., 2013; Quaedflieg et al., 2013, 2015; Capello and Markus, 2014), pulse rate (PR) is yet to be investigated as a measure of autonomic functioning in response to the MAST, and as alluded to above, no other study has measured the anticipatory responses prior to the MAST. Importantly, for a lab stress test to be useful for research into cognition and attention under acute stress, a reliable stress response must last some time to allow subsequent performance measurements of participants. Fortunately, Bos et al. (2014) established that BP was reliably increased immediately after the MAST procedure, though it had returned to baseline by 20 min post-MAST, whilst Smeets et al. (2012) only measured BP at 5 min post-test. Smeets et al. (2012) also demonstrated cortisol levels to be elevated after 30 min, whilst alpha-amylase levels were no longer significantly elevated after 10 min. Thus, we sought to investigate both anticipatory effects on BP and heart rate following hearing instructions, and also for the first 30 min following the conclusion of the procedure. Anticipatory responses (i.e., baseline vs. subsequent) were assessed using a repeated measures design in order to quantify the duration of the SAM responses. In addition, state anxiety before and after the MAST procedure was assessed. Finally, individual differences in pre-existing anxiety and depressive symptoms were also explored as possible contributors to the abovementioned reactions to a moderate, acute stress.

## Materials and Methods

### Participants

A total of 60 adults with a mean age of 23.6 years (*SD* = 4.4) participated in the current study. This sample included 48 women and 12 men. Participants were recruited through advertisements at La Trobe University, and online forums that stated that the experiment was exploring people’s resilience to physical and mental challenges. Eligibility was assessed using an online screening questionnaire. Exclusion criteria was adopted from Smeets et al. (2012) and included cardiovascular diseases, severe physical illnesses (e.g., fibromyalgia), hypertension, endocrine disorders, current, or lifetime psychopathology, substance abuse, heavy smoking (>10 cigarettes/day) or being on any kind of medication known to affect the HPA axis. This project was carried out in accordance with the recommendations of the La Trobe University Faculty of Science Technology & Engineering Human Ethics Committee, which reviewed and approved the study. Written informed consent was provided by all participants. All participants gave written informed consent in accordance with the Declaration of Helsinki, and received a small financial reward in the form of a voucher after completing the testing at La Trobe University.

### Maastricht Acute Stress Test

The MAST (Smeets et al., 2012) begins with a 5 min preparation phase to allow the participant to read the instructions for the upcoming task (on a PowerPoint presentation). In the following 10 min acute stress phase, physical stress (e.g., cold induced pain) is combined with unpredictability, uncontrollability, and social evaluation in a mental arithmetic task.

Participants were informed that there would be alternating trials of immersing their hand into ice-cold water (maintained at 2°C by use of a Huber Unichiller high precision thermoregulator), and engaging in a mental arithmetic task (counting aloud backward from 2043 in steps of 17). They were told that the duration of these trials would be randomly chosen by the computer to last between 45 and 90 s and used their non-dominant hand (56 participants were right-handed). In between the hand immersion trials, participants resumed the counting task while they rested their arm on a towel beside the water bath. If they made a mistake with accuracy or did not give a response within 5 s, negative feedback was given by the experimenter and the participant had to start again at 2043. Participants were also informed they would be video-recorded so as to later analyze their facial expressions.

In reality, the duration of all trials were pre-determined with the same protocol used for all participants. Five hand immersion trials (HI) were alternated with four mental arithmetic trials (MA) in the following order and length, HI (90 s), MA (45 s), HI (60 s), MA (60 s), HI (60 s), MA (90 s), HI (90 s), MA (45 s), HI (60 s). Participants were unaware of the number of trials and the total duration of the stress phase.

### Cardiovascular and Subjective Stress Responses

#### Physiological Measures

Systolic and diastolic blood pressure, as well as PR (as a proxy for direct heart rate measurements) were measured using an iHealth BP7. This device is an automated wrist oscillometric BP monitoring device that has been validated against mercury sphygmomanometer measurements from two observers, and reported a mean ± SD device-observer difference of -0.7 ± 6.9 mmHg for SBP, and -1.0 ± 5.1 mmHg for DPB (Wang et al., 2014), and has TGA approval in Australia (also FDA in the US, C.E in Europe, and Health Canada approval). SBP, DBP, and PR were all measured at six time points for each participant. A baseline measure was taken prior to the MAST [T(baseline)], immediately after instruction but prior to the MAST [T(post-instructions)], as well as immediately after [T(+00)] and 10, 20, and 30 min post-MAST completion [T(+10), T(+20), and T(+30) respectively]. Measurements were taken from the opposite arm to that used for cold water immersion.

#### Subjective Measures

Changes in anxiety levels for each participant were measured using repeated administrations of the State-Trait Anxiety Inventory (STAI-Y) (Spielberger, 1983). The STAI-Y consists of two separate 20-item self-report scales that measure state and trait anxiety. Both the STAI-Y state and trait scales were administered prior to the MAST (including prior to MAST instructions), and only the state anxiety scale was re-administered immediately after the stress protocol. The state anxiety scale asks the participant to indicate ‘*how you feel right now, at this moment,’* whereas the trait anxiety scale asks ‘*how you generally feel.’* Both state and trait anxiety were rated using a Likert scale ranging between 1 and 4 (1 = not at all; 4 = very much so).

The Depression Anxiety Stress Scales (DASS-21) is a short form of the original 42-item self-report measure of depression, anxiety, and stress developed by Lovibond and Lovibond (1995). The DASS-21 was administered as a baseline measure with participants asked to indicate how much each statement applied to them over the past week. The 21-item were all rated on a four-point scale (0 = Did not apply to me at all – NEVER; 3 = Applied to me very much, or most of the time – ALMOST ALWAYS).

### Procedures

Participants first completed an online questionnaire including questions relating to basic demographics and exclusion criteria, and were subsequently invited to complete individual testing at La Trobe University. On arrival written consent was obtained, followed by baseline measures of subjective (STAI-Y and DASS-21) and physiological (SBP, DBP, PR) anxiety and stress. A series of computer-based visual perception tasks not associated with this study were carried out for approximately 40 min. (These visual tasks were designed to assess basic visual processing such as object recognition and were not cognitively demanding.) Participants then completed the 15 min MAST protocol (preparation phase, hand immersion, and mental arithmetic trials), and immediately after (but before being told the MAST procedure was finished), SBP, DBP, PR, and the State anxiety subscale of the STAI-Y were all measured (with the SBP, DBP, and PR measures then repeated approximately 10, 20, and 30 min post-MAST).

### Data Analyses

Of the total 60 participants tested, three did not finish the experiment due to not being willing to tolerate the MAST procedure leaving a sample of 57. Outliers were defined as data lying greater than three interquartile ranges beyond the 25th and 75th percentiles, however, there were no such extreme outliers. Primary analyses investigating the effects of the MAST included only healthy participants as indicated by DASS-21 scores within the Normal – Moderate range. As a result, five participants were not included in this analysis (*n* = 52). Secondary analyses aimed to explore individual differences in depression, anxiety, and stress levels through parametric correlations with physiological responses to the MAST, and so all participants were included in this analysis regardless of their DASS-21 score (*n* = 57). The data was checked for non-normality using Q–Q plots and Shapiro–Wilks tests of normality. The Expectation-Minimisation method was used to manage missing data in the current data set (Schafer, 1997; Schafer and Olsen, 1998).

The primary analyses included one-way repeated measures ANOVA’s that were conducted to evaluate the impact of the MAST procedure on SBP and DBP, as well as PR over the six time points [T(baseline), T(post-instructions), T(+00), T(+10), T(+20), and T(+30)] in a healthy population. Where the assumption of sphericity was not met, Greenhouse–Geisser corrections were applied. *Post hoc* analyses were conducted using Tukey HSD multiple comparisons to determine which time points were significantly different, with alpha set at 0.05. State anxiety (STAI-Y scores) before and after the MAST was analyzed using a paired-samples *t*-test. Secondary analyses were performed using parametric correlations on the larger sample (with participants scoring high on the DASS re-included) (*n* = 57) to determine if there is a relationship between levels of depression, anxiety, and stress (DASS-21) on the one hand, and both BP- and PR- reactivity, defined as change scores between baseline and immediately following the MAST.

Due to the uneven ratio of males to females, we re-ran the main analyses for females only (*n* = 41). Repeated measures ANOVA analyses for SBP [*F*(3.75,150.21) = 16.00, *p* < 0.001, $\eta_{p}^{2}$ = 0.29], DPB [*F*(3.72,148.94) = 240.14, *p* < 0.001, $\eta_{p}^{2}$ = 0.86], PR [*F*(4.01,163.52) = 2.43, *p* = 0.048, $\eta_{p}^{2}$ = 0.06] and paired-sample *t*-test for State anxiety [*t*(40) = 11.60, *p* < 0.001] all established substantially similar patterns of results (see Supplementary Figure 1) as for analyses of males and females together described in the Section “Results”.

## Results

**FIGURE 1:**
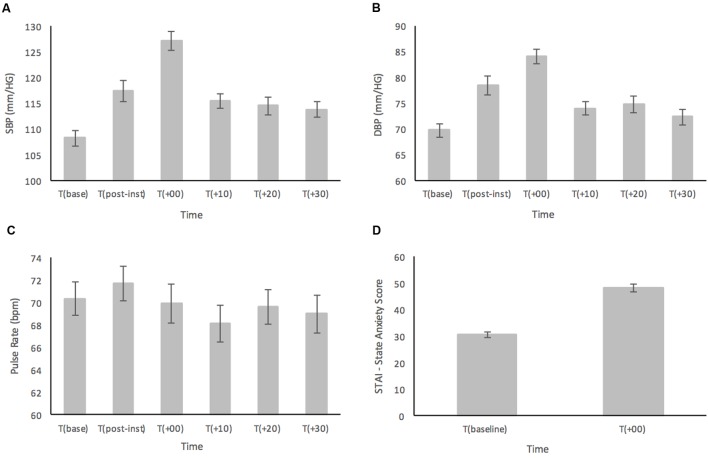
**(A)** Systolic blood pressure (SBP), **(B)** diastolic blood pressure (DBP), **(C)** pulse rate (PR), and **(D)** subjective ratings based on the STAI-Y, prior to and in response to the MAST in a healthy sample (*n* = 52). Error bars represent ± standard error of the mean.

Results for SBP are shown in **Figure 1A**. For the primary analyses on the healthy participants, ANOVA results showed a main effect for Time [*F*(4.05,206.46) = 23.12; *p* < 0.001, $\eta_{p}^{2}$ = 0.312] (see **Figure 1A**). *Post hoc* comparisons demonstrated that SBP at T(base) was significantly lower compared to all time points (all *p*s < 0.028) except at T(+30) (*p* = 0.08). At T(post-instructions), SBP had significantly increased from baseline (*p* < 0.001), yet it was still significantly lower than T(+00) (*p* < 0.001), and showed no significant difference compared to any other time point (all *p*s > 0.474). The peak in SBP was reached immediately after the MAST at T(+00) and was significantly higher than all other time points (all *p*s < 0.001). There were no significant differences for SBP between T(+10), T(+20), and T(+30) (all *p*s > 0.957).

**FIGURE 2:**
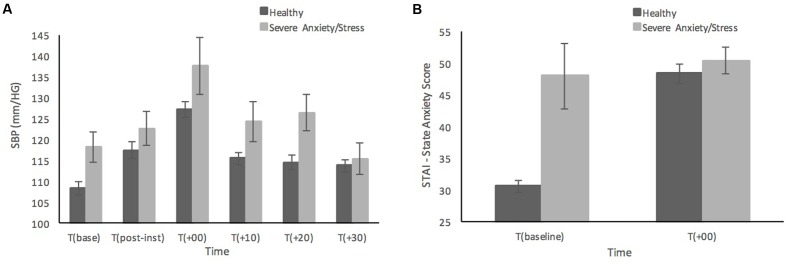
**Systolic blood pressure** **(A)** and subjective state anxiety based on the STAI-Y **(B)** prior to, and in response to the MAST in healthy sample (*n* = 52) compared to individuals with severe anxiety and stress (*n* = 5). Error bars represent ± standard error of the mean.

Similarly, analyses of DBP (**Figure 1B**) showed a main effect for Time [*F*(3.94,200.86) = 23.16; *p* < 0.001, $\eta_{p}^{2}$ = 0.312] (see **Figure 2**). In the *post hoc* analyses, DBP was significantly higher at T(post-instructions), T(+00) and T(+20) compared to T(base) (all *p*s < 0.036). However, DBP was not significantly higher at T(+10) (*p* = 0.115) and T(+30) (*p* = 0.641) when compared with T(base). DBP at T(post-instructions) was significantly higher compared to T(+30) (*p* < 0.005), but not compared to T(+10) and T(+20) (*p*s > 0.079). DBP similarly reached a peak at T(+00), being significantly higher than all other time points (all *p*s < 0.017). There were no significant differences for DBP between T(+10), T(+20), and T(+30) (all *p*s > 0.685).

As displayed in **Figure 1C**, PR showed a relatively stable pattern with large variance across the time points measured. There was a significant main effect for Time [*F*(5,255) = 2.47; *p* = 0.033, $\eta_{p}^{2}$ = 0.046]. Interestingly, PR reached the highest point at T(post-instructions), and not at T(+00) as was the case for both SBP and DBP though this was not significant. *Post hoc* analyses showed that the only significant result indicated that PR was higher at T(post-instructions) compared to T(+10) (*p* = 0.016).

Subjective ratings of state anxiety as measured by the STAI-Y were subjected to a paired samples *t*-test and demonstrated a large significant increase in anxiety levels from T(baseline) (*M* = 30.65, *SD* = 6.93) compared to immediately following the MAST at T(+00) (*M* = 48.38, *SD* = 10.78), *t*(51) = 12.23, *p* < 0.001 (two-tailed) (see **Figure 1D**).

### Correlations between Physiological Reactivity and Psychological Measures

Physiological and psychological reactivity were defined as change scores between baseline and immediately following the MAST. A bivariate Pearson’s correlation showed a moderate positive correlation between DASS-Dep and SBP reactivity [*r*(57) = 0.336, *p* = 0.011], and a small positive correlation between DASS-Stress and SBP reactivity [*r*(57) = 0.267, *p* = 0.045]. There was also a moderate negative correlation between STAI-State Reactivity and DASS-Stress [*r*(57) = -0.352, *p* = 0.007] indicating that participants with higher self-reported stress ratings showed a smaller degree of change in state anxiety scores pre and post the acute stress intervention (see **Table 1** for all correlations between DASS subscales and physiological and psychological reactivity scores).

**Table 1:** Pearson correlation coefficients (r) for physiological reactivity (SBP, DBP, PR) and psychological reactivity (STAI-State) with DASS scores.

	DASS-Dep	DASS-Anx	DASS-stress
SBP reactivity	0.336^∗^	0.052	0.267^∗^
DBP reactivity	0.215	0.110	0.172
PR reactivity	0.043	0.147	-0.083
STAI-state reactivity	-0.152	-0.237	-0.352^∗^


*^∗^correlation significant at the 0.05 level (two-tailed) (*n* = 57).*

**Figure 2** shows the differences in SBP and state anxiety scores of the five participants who scored in the ‘Severe’ or ‘Extremely Severe’ range on the DASS-21 Anxiety and Stress subscales compared to remaining sample of healthy individuals. Those with severe anxiety and stress are shown to have relatively consistently higher SBP than healthy participants across time points, although at 30 min post-MAST the individuals with severe anxiety and stress have recovered to a similar SBP level as the healthy sample (see **Figure 2A**). It is not surprising that the individuals with severe anxiety and stress reported much higher baseline levels of state anxiety compared to the healthy sample (see **Figure 2B**). Although interestingly both groups reported similar levels of state anxiety immediately after the MAST procedure, those with severe anxiety and stress show much smaller increases in state anxiety from baseline, compared to the significant increase in state anxiety from baseline in healthy participants.

In the intervening 30 min between finishing the MAST and taking the physiological measurements, participants completed further computer-based tasks with low cognitive demand (again not associated with this study), which meant physiological measures were always taken after similar intervals but not always at exactly the prescribed times. Correlation analyses were run to determine if the timing variation and any outliers impacted on the overall results. Both SBP and DBP, as well as PR, did not correlate with the variation in timing around each time point, and was thus assumed to not impact the above-described results.

## Discussion

The current study investigated both autonomic changes, and subjective levels of anxiety, in response to the MAST, in order to validate and broaden some of the previous research findings of Smeets et al. (2012) and Meyer et al. (2013) in particular. Individual differences in self-reported levels of psychopathology were examined as a correlate with physiological reactivity to stress. Overall our results showed that in healthy individuals SBP was elevated above baseline for up to 30 min post-MAST, however DBP had already returned to baseline by 10 min post-MAST. There was also a significant elevation above baseline for both SBP and DBP after hearing only the instructions of the MAST (i.e., in anticipation of the forthcoming procedure), albeit not as high as post-MAST measurements. Measurements of PR showed limited variability across time points and was thus inconclusive. Self-reported state anxiety increased significantly immediately following the MAST compared to baseline levels, and higher self-reported levels of depression and stress was also likely to be accompanied by larger increases in SBP in response to the MAST.

### Physiological Responses to the MAST

Results for both SBP and DBP in the healthy sample indicate that the MAST is capable of eliciting a strong autonomic stress response (i.e., SAM axis) immediately following the procedure. Consistent with previous findings on BP reactivity in response to stress tests, including the MAST (Smeets et al., 2012), the current results showed there was a larger increase in SBP compared to DBP. Only one other study has measured BP beyond 5 min post-MAST, with Bos et al. (2014) finding SBP was significantly elevated immediately after the procedure, though these levels had returned to baseline by 20 min. In contrast the current results suggested SBP was reduced compared with measurements immediately following the procedure, though they remained significantly elevated for up to 20 min post-MAST, and possibly as long as 30 min given BP is very similar across the 10, 20, and 30 min time points. These results hence are consistent with the timeframe of elevated cortisol responses reported by Smeets et al. (2012) and highlight the suitability of testing cognitive or behavioral performance in a time period following the MAST protocol. In the current study, the response pattern for DBP was quite different to that seen for SBP. By 10 min post-MAST, DBP had recovered to be non-significantly higher than baseline levels. At 20 min post-MAST, DPB was marginally significantly higher than baseline, though this was not significantly higher when compared to the 10 or 30 min post-MAST measures. This appears to indicate that DBP recovers to baseline levels faster than SBP, and may implicate SBP as a key component of longer-term responses to acute stress. Although different measurement times were utilized by Bos et al. (2014), a similar broad pattern was also established for DBP, with levels returning to baseline levels by 20 min. Previous research has suggested that SBP is more commonly implicated in stress outcomes compared to DBP, with, for example, significant SBP, and not DBP, reactivity following moderate acute stress (Yuenyongchaiwat et al., 2016), and SBP, but not DBP, associated with increased cardiovascular risk across a 23-year longitudinal study (Hao et al., 2017). However, the reasons underlying this pattern remain unclear. It is also important to note that despite SBP remaining higher at 10 and 20 min post-MAST compared to baseline, the average difference compared to baseline was 5.9 mmHg (5.2%), with a clear peak in SBP immediately after the MAST (127 mmHg), and which lasted less than 10 min.

When considering the clinical significance of the elevations in BP, the classification stages of high BP may serve as a useful comparison. Pre-Hypertension is considered to be between 120 and 139 for systolic and 80–89 for DBP, while High Blood Pressure Stage 1 is 140–159 for systolic and 90–99 for DBP (National High Blood Pressure Education Program, 2004). Our results indicate that immediately following the MAST participants reach a level comparable to the Pre-Hypertensive range in both SBP and DBP, while they dropped back into normal range thereafter. This may indicate that after 10 min post-MAST, BP may not have persistent clinically significant effects on cognition and behavior. However, these stages of high BP may be quite different to acute stress induced BP increases that can cause cognitive and behavioral impairments, as seen in a study on BP response to exam stress and the impact on exam performance (Hughes, 2007). Hughes found an average increase in SBP of 13.8 and 9 mmHg for DBP was associated with better performance on the exam, suggesting this level of change was adaptive and helpful. The data in the current study showed strong changes in SBP immediately after the MAST (19 mmHg increase from baseline). Cognitive testing following the MAST is needed to determine if this degree of increased BP would be adaptive and advantageous, or if it would instead lead to cognitive and behavioral impairments as expected of a lab stress test. Meyer et al. (2013) have provided some insight into the cognitive effects of increased cortisol following the MAST procedure. They found that individuals with increased cortisol showed improved performance on a task requiring implicit spatial memory processing, whilst those that had no change in cortisol showed worse performance. When looking at SBP at 10–20 min post-MAST procedure, levels are only raised by 5.9 mmHg and it could be surmised that this difference may not be clinically significant in terms of affecting cognitive and behavioral outcomes. Further research testing cognition and attention following the MAST will need to confirm this.

The BP results in the current study also indicate that there is an anticipatory effect of the MAST; the instructions alone were strong enough to elicit substantial SBP and DBP increases. Given the instructions simply required cognitive understanding of the upcoming task and was therefore psychological in nature, it would seem that the MAST instructions alone can be a considerable psychological stressor. BP has seldom been studied in anticipation to stress, but our results do support previous findings that SBP increased in anticipation of a physical stressor (blood test) (Marshall et al., 2002). However, we also showed the same anticipation effect in DBP, while Marshall et al. (2002) did not. Given the MAST consists of both physiological and psychological stressors, it is unknown if one or both components are eliciting the anticipatory BP increases, and future research should consider the impact of the type of task on anticipatory responses.

Results for the PR data in the current study shows limited change across the time points prior to and in response to the MAST. In contrast to the BP results, there was no anticipation effect on PR after the instruction of the MAST, and no significant increases above baseline after the MAST procedure were detected. This limited PR reactivity could indicate an adaptive response to acute stress, with a quick recovery as is expected for the autonomic system. This natural flexibility of the ANS to transition between high and low states of arousal, and to rapidly vary heart rate, means there is a relatively small timeframe to capture this process, but is arguably a much more ecological and healthy adaptive response. Therefore, direct heart rate measurements from ECG, with continuous recordings of heart rate is desirable to enable a calculation of acute heart rate variability that can accurately reflect autonomic flexibility, may be more accurate than PR variability (Schafer and Vagedes, 2013) and provide a more in depth understanding of heart rate responses whilst anticipating, experiencing, and following acute stress.

### Subjective Responses to the MAST

Notably, the current results demonstrated the MAST is capable of eliciting strong increases in state anxiety (STAI-Y). Although the post-MAST state anxiety measure was technically taken at the completion of the procedure, participants were told this was a rest period and so believed the procedure would be continuing. Therefore, the post-MAST measure in the current study likely reflects state anxiety levels experienced during the procedure rather than after it. Hellhammer and Schubert (2012) have recently shown subjective ratings of distress were lower immediately after the stress compared to during the stress. This point should be taken into account when considering the significant elevations of state anxiety in the current study, as it is unclear exactly how long this effect may last once participants knew the MAST was finished. Although significant increases in subjective psychological distress have been found immediately after the MAST, there was either no further follow up (Smeets et al., 2012) or distress ratings had significantly declined after 40 min post-MAST (Meyer et al., 2013). Although state anxiety was not measured in anticipation of the MAST, recent research has shown there are often anticipation effects of increased subjective psychological stress as demonstrated using the Primary Appraisal Secondary Appraisal (PASA) questionnaire in response to a range of stress test protocols (Skoluda et al., 2015). This highlights the importance of investigating subjective psychological stress at a baseline level, in anticipation of the stress, as well as during and after the stressor.

### Correlates of Psychological Measures with Stress Responses

Investigations into psychological mediators of physiological reactivity to the MAST revealed that levels of depression and stress were positively correlated with SBP reactivity. This supports the findings of Kibler and Ma’s (2004) review which found depressive symptoms (in clinical and non-clinical samples) were positively associated with BP and heart rate responses across a range of stress tests (e.g., Stroop, speech). However, our results were not in line with the findings from a more recent review conducted by Chida and Hamer (2008), who suggest that depressed mood (in subclinical samples) was one of the significant psychosocial factors negatively associated with cardiovascular reactivity (especially SBP) during cognitive, emotional and interpersonal acute stressors. Although general life stress was not associated with cardiovascular reactivity, it was a significant factor for poor cardiovascular recovery (Chida and Hamer, 2008). Perhaps more surprisingly from the current data, anxiety levels as measured on the DASS-21 did not seem to predict physiological responses to the MAST. Although the small number of individuals with severe anxiety and stress demonstrated higher overall SBP, changes in BP reactivity to the MAST seems to be more related to stress than anxiety. This differs from the results of de Rooij et al. (2010) who found that with increased self-reported anxiety symptoms, SBP and heart rate reactivity decreased. Such discrepancies may be due to the different stress tests and anxiety measures used. de Rooij et al. (2010) study involved three 5 min stress tests that were mental or social stressors and measured anxiety on the Hospital Anxiety Depression Scale (HADS), whereas the current study used the MAST that has physical, mental and social stressor elements, and measured anxiety on the DASS-21. Nonetheless, it appears that general depression and stress levels are important psychological mediators in the physiological response to acute stress. Stress, as measured on the DASS-21 which purportedly targets subjective symptoms of anxiety (Lovibond and Lovibond, 1995), was the only factor to correlate with state anxiety reactivity. Anxiety, as measured on the DASS-21 which purportedly targets the physiological arousal of anxiety (Lovibond and Lovibond, 1995), did not affect state anxiety reactivity (STAI-Y) to the MAST. These results suggest that state-anxiety reactivity is more strongly affected by the subjective experience of anxiety rather than self-reported physiological symptoms. The current study used an individual differences approach rather than seeking to understand the effects of clinical anxiety and depression on acute stress responses, however, further investigations about how both clinical and subclinical anxiety and stress predict acute stress responses is needed.

One limitation of the current study should be noted. The unequal gender ratio, with more females than males participating, limits the potential to make conclusions about gender differences in response to the MAST procedure. In fact, conclusions from the current study are mostly applicable to healthy younger females. As shown in the Supplementary Figure 1, males appeared to show overall higher levels of SBP which is consistent with previous research (see Kajantie and Phillips, 2006 for review), however, it should also be noted that males and females may respond differently to acute stress (Kirschbaum et al., 1999). In particular, menstrual cycle, which was not examined here, can influence the extent of physiological responses to physical stress (Tersman et al., 1991). As a consequence, increased variability in the female data is possible, although it is noteworthy that a clear and significant pattern of results was still established. A second limitation, relates to the fact that participants completed some visual computer tasks prior to measuring their baseline PR and BP, as well as in between the post-MAST PR and BP measures. These visual computer tasks were not designed to be stressful within themselves, however, they may have caused unforeseen changes in PR and BP in some participants. We argue that this appears unlikely given that counterbalancing the order of visual tasks revealed no differences in BP or heart rate measures. A third limitation of the study is the lack of temporal resolution from the BP and PR monitoring, which did not allow for continual readings and therefore the changes occurring immediately prior to, during, and following the MAST, are not known. However, although of interest, our main interest was not the development of stress responses *during* the MAST protocol, but the extent and longevity of the stress response following the procedure as this will provide an indication of the time-frame for researchers to examine the impact of acute stress on cognition and behavior immediately following the MAST.

## Conclusion

Our findings show that the MAST is able to induce significant autonomic responses with regards to SBP and DBP, but not PR. A significant increase in SBP and DBP was seen immediately following the MAST, with a smaller yet still significant elevation in SBP lasting between 20 and 30 min post-MAST procedure. This consists of a longer timeframe than previously reported (Smeets et al., 2012; Bos et al., 2014). Significant elevations in both SBP and DBP were apparent after hearing only the instructions, suggesting an anticipatory physiological stress response to the MAST, which has not been measured previously. Overall, there was limited variability of PR in response to the MAST and this likely reflects the adaptive ANS process that suppresses heart rate (for which PR served as a proxy in the current study). Therefore, it will be important for future studies to measure PR, or preferably heart rate directly and continuously to get a measure of heart rate variability. The current findings also replicate previous research, demonstrating that the MAST is capable of eliciting strong increases in subjective levels of state anxiety (Smeets et al., 2012). This important insight into the link between acute stress and feelings of anxiety leads to further questions of how psychological responses to stress may lead to maladaptive anxiety disorders or perhaps other psychopathology. Future research should endeavor to more thoroughly measure subjective ratings of stress or anxiety and explore the interaction between the psychological and physiological responses to an acute stressor. Initial insights into the physiological responses to the MAST suggest individual differences in depression and stress symptoms predict SBP reactivity. Overall, this study has demonstrated that the MAST is an efficient stress test protocol for inducing increases in BP and subjective levels of state anxiety in a healthy population. Although the stress test seems to evoke similar responses in individuals with severe anxiety and stress, there is a need for further investigations in clinical populations.

## Author Contributions

AS contributed to the design of the study; acquisition, analysis and interpretation of the data; writing, drafting and final approval of the version to be published. RL contributed to the conception and design of the study; analysis and interpretation of the data; drafting and final approval of the version to be published. SC contributed to the conception of the study; interpretation of the data; drafting and final approval of the version to be published.

## Conflict of Interest Statement

The authors declare that the research was conducted in the absence of any commercial or financial relationships that could be construed as a potential conflict of interest.
