# Supplementary material for: The Maastricht Acute Stress Test (MAST): Physiological and Subjective Responses in Anticipation, and Post-stress
Source: Front Psychol. 2017 Apr 19;8:567. doi: 10.3389/fpsyg.2017.00567 (PMC5395611; doi:10.3389/fpsyg.2017.00567)
Supplement: Supplementary file 1 [file Data_Sheet_1.PDF]

frontiers

Supplementary Material

The Maastricht Acute Stress Test (MAST) as a Physiological and Subjective Measure of Stress Responses

Alexandra L. Shilton, Robin Laycock, and Sheila G. Crewther*

*** Correspondence:** Corresponding Author: s.crewther@latrobe.edu.au

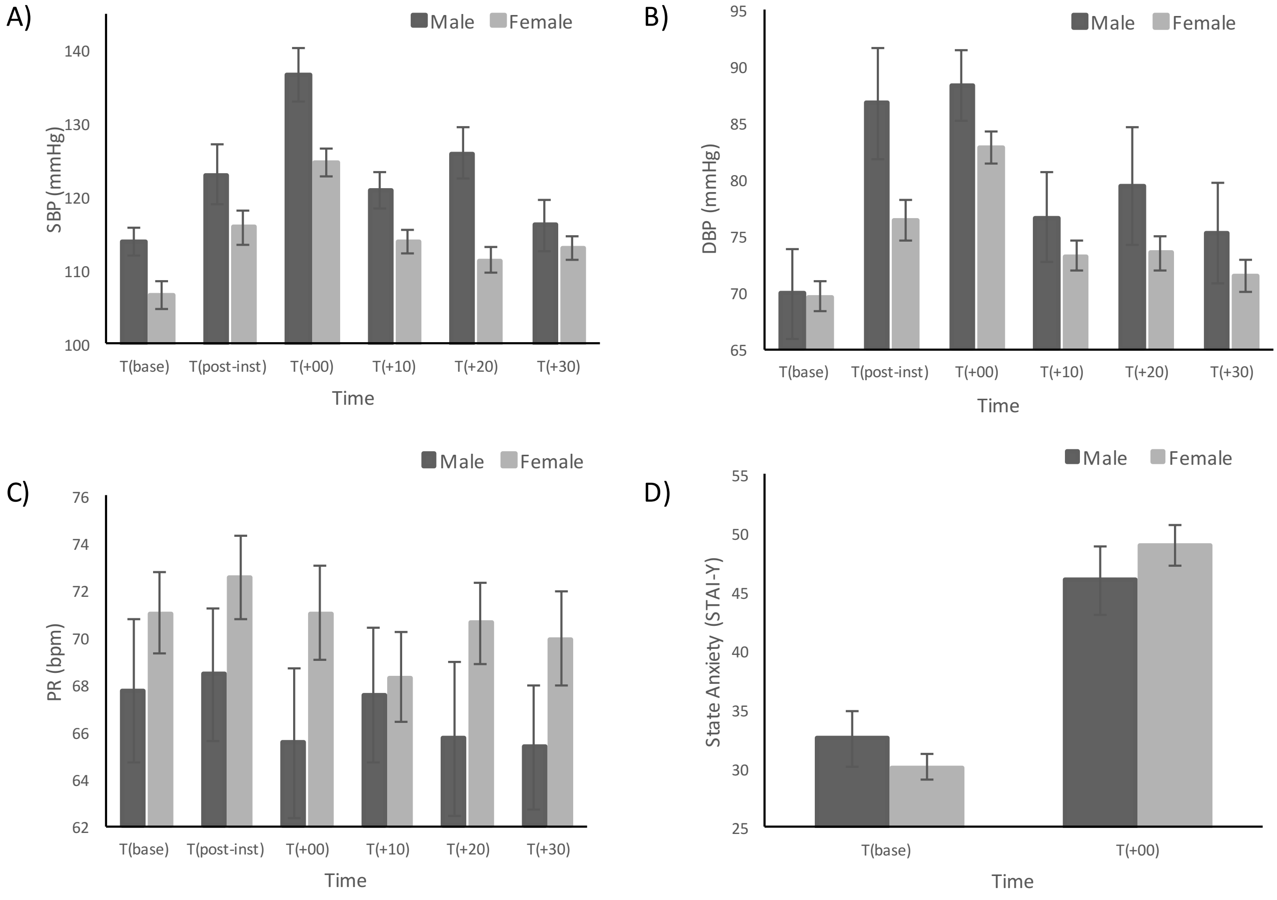

Supplementary Figure 1. SBP (A), DBP (B), PR (C), and State Anxiety (STAI-Y) (D) before and after the MAST procedure in males and females (n=52). Error bars represent ± standard error of the mean.
